# An investigation on glutathione derived from spinach and red cabbage leaves and their effects of adding to meat patties

**DOI:** 10.1016/j.sjbs.2023.103632

**Published:** 2023-03-31

**Authors:** Anfal Alwan AL-Temimi, Sawsan A. Al-Hilifi, Aum-El-bashar AL-Mossawi

**Affiliations:** Department of Food Science, College of Agriculture, University of Basrah, Basrah 61004, Iraq

**Keywords:** Leaf producing vegetables, Glutathione, Antioxidants, Food formulations, Food industry, HPLC

## Abstract

Plants that produce leaves have been cultivated by humans for thousands of years because of the benefits they provide in terms of food and other necessities. Because of their high nutritional value and key phyto-components like glutathione, Leaf producing vegetables (LPVs) are being studied for their potential uses and health benefits. As a result, the focus of this study was using efficient methods for isolating and identifying glutathione from spinach and red cabbage. Glutathione was extracted using three extraction solvents: water (100%), ethanol (100%), and a combination of ethanol and water (30% and 70%, respectively) by volume (v/v), while separation was accomplished using ultrafiltration equipment. In our investigation, the best extraction solvent was a mixture of ethanol and water at a ratio of 30:70% (v/v), which extracted 951 µg/g glutathione. The antioxidant activity of plant leaf extract was measured using DPPH, with butylated hydroxytoluene serving as a comparative standard. Identification and characterization of glutathione from plant leaf extracts were revealed by thin-layer chromatography (TLC), ultraviolet–visible (UV–Vis) spectrophotometry studies, Fourier transform infrared (FTIR) spectroscopy, and high-performance liquid chromatography (HPLC). In addition, the physical and chemical properties (pH, water holding capacity, extracted liquid volume, peroxide value, free fatty acids, and thiobarbituric acid) of meat patties prepared with three different concentrations of determined glutathione were tested for susceptibility to preservation during 10 days of refrigeration at 4 ± 1 °C. The findings of the current study provide vast prospects for subsequent research to researchers and scientists that the glutathione obtained from leaf extract has no toxicity that might be applied to developed functional foods and other food formulations. Because foods containing plant-derived glutathione improve health, biological function, and food spoilage. It may be utilized as high-quality antioxidants that are safe and non-toxic. Furthermore, glutathione preserves food quality and prevents oxidation.

## 1 Introduction

The agricultural sector is essential not only for ensuring the long-term safety of the nation’s food supply but also for combating poverty (Verma et al., 2014, Goyal and Verma, 2018, Verma et al., 2018a, McDaniel and Jadeja, 2019). It is widely known that agriculture plays one of the most important roles in the economy across all nations, but especially in economies of those nations that are still developing (Soubbotina and Sheram, 2000, Verma et al., 2014, Goyal and Verma, 2018). The agricultural industry is considered to be the foundation of the world economy. The growing of vegetables that produce leaves is known as leaf producing vegetables (LPVs), also an essential component of the global agricultural economy everywhere in the globe (Goyal and Verma, 2018, Verma et al., 2018b). In addition, LPVs have been a staple of the human diet since ancient times since it was believed that they offered a variety of advantages to one’s health (Verma et al., 2018b, Verma et al., 2020, Sarkar et al., 2022b, Al-Hilifi et al., 2022a). In later years, it was revealed that LPVs had an exceptionally high nutritional content and may be cultivated to provide food and other advantages to humans (Verma et al., 2018b, Verma et al., 2020, Al-Hilifi et al., 2022b). In addition to its use as a food source, LPVs have been shown in several studies to reduce the risk of numerous different forms of degenerative illness, including those related to carcinogenesis, brain function, cardiovascular health, diabetes, heart disease, hypertension, and inflammation (Verma et al., 2018b). Minerals like iron (Fe), calcium (Ca), and zinc (Zn) are found in particularly high concentrations in LPVs. In addition to that, they have a high concentration of vitamins such as beta(β)-carotene, and vitamins E, K, B, and C (Verma et al., 2018b, Sarkar et al., 2022b). Furthermore, some of the anti-nutritional components (like oxalate, phytate and glucosinolates) that are found in LPVs can be mitigated if the crops are produced well and processed appropriately before ingestion (Verma et al., 2018b, Verma et al., 2020, Verma et al., 2021b, Sarkar et al., 2022b).

Spinach (*Spinacia oleracea* L.) and red cabbage (*Brassica oleracea* L. var. capitata), both of which are considered as LPVs, are just two examples of the many different types of LPV plants that can be found in the various nations of the globe (Sarkar and Rakshit, 2017, Phahlane et al., 2018, Song et al., 2018, Verma et al., 2018b, Gutierrez et al., 2019, Sarkar et al., 2022a, Sarkar et al., 2022b). Spinach is a member of the family Amaranthaceae; it grows as an annual (or occasionally biennial) plant that is often considered to be the leafy green crop with the second highest nutritional value in the world (Sarkar and Rakshit, 2017, Gutierrez et al., 2019, Sarkar et al., 2022b). In terms of its nutritional value, spinach is an excellent source of vitamins A (9380 IU/100 g of edible portion), K1 (483 mg/100 g of edible portion), and folate (194 mg/100 g of edible portion), all of which are nutrients that are necessary for the human body to carry out a variety of metabolic processes (Gutierrez et al., 2019, USDA, 2019). Spinach possesses comparatively one of the greatest oxygen radical absorbance capacities (ORAC) of any vegetable, which not only makes it an exceptional source of antioxidants but also gives it one of the highest ORAC values overall (Ou et al., 2002, Roberts and Moreau, 2016, Gutierrez et al., 2019). Spinach has several health advantages, including protection against cancer and obesity, protection against damage caused by macromolecular oxidation, and protection against age-related macular degeneration (Roberts and Moreau, 2016, Verma et al., 2018b, Verma et al., 2020, Gutierrez et al., 2019, Sarkar et al., 2022b). On the other hand, red cabbage, a member of the family Brassicaceae that is popularly regarded as the “dream food of the modern multitasker,” has recently attracted greater attention all around the world (Draghici et al., 2013, Sarkar and Rakshit, 2017, Verma et al., 2018b). This extraordinary and nutrient-dense vegetable is a wonderful source of health-promoting components that can help strengthen our immune system (Huang et al., 2016, Mizgier et al., 2016, Phahlane et al., 2018, Verma et al., 2018b, Sarkar et al., 2022a). The selection of crops is another significant weapon in our armory against degenerative illnesses, which have wreaked havoc on the livelihoods of humans and brought the world economy to its knees. Red cabbage is an excellent dietary supplement for the mitigation of cancer disease, cardiovascular disease, diabetes disease, obesity, and oxidative stress because it contains a high concentration of antioxidants, phenols, anthocyanins, carotenoids, glucosinolates, minerals (Ca, Fe, K, Mg, and Mn), and vitamins (A, E, K, and C) (Leja et al., 2010, Draghici et al., 2013, Foyer and Noctor, 2011, Phahlane et al., 2018, Song et al., 2018, Kaur et al., 2021, Gao et al., 2022, Sarkar et al., 2022a). Because of this, it may be consumed raw in the form of microgreens and salads, as well as cooked like any other vegetable (Draghici et al., 2013, Foyer and Noctor, 2011, Sarkar and Rakshit, 2017, Verma et al., 2018b, Sarkar et al., 2022a, Rizvi et al., 2022). Microgreen forms of the plant are significantly more nutritious than fully developed forms of this plant. They have ∼260 times more β-carotene, >40 times more vitamin E, and 2.4–6.0 times more vitamin C (Huang et al., 2016, Sarkar et al., 2022a, Rizvi et al., 2022). Because of the high concentrations of anthocyanins (40–188 mg Cy 3-glcE 100 g^−1^ FW) that are present in red cabbage, it is possible to extract natural dyes from this vegetable, which may then be used to color food product (Mizgier et al., 2016, Verma et al., 2018b, Gao et al., 2022, Sarkar et al., 2022a). In comparison to white cabbage, red cabbage has a higher content of beneficial nutrients including antioxidants, vitamins, and minerals (Leja et al., 2010, Sarkar and Rakshit, 2017, Sarkar et al., 2022a).

The LPVs, such as spinach and red cabbage, are essential to a diet that is healthy since these vegetables can treat medical conditions owing to the high bioactive and nutritional potential that they carry (Leja et al., 2010, Roberts and Moreau, 2016, Sarkar and Rakshit, 2017, Verma et al., 2018b, McDaniel and Jadeja, 2019, USDA, 2019, Verma et al., 2020, Banwo et al., 2021, Sarkar et al., 2022b). The nutritional value and bioactive properties of LPVs may be drastically changed by the processing methods used to prepare them (Verma et al., 2018b, Srivastav et al., 2020, Verma et al., 2020, Singh et al., 2021, Verma and Thakur, 2021, Sarkar et al., 2022b). Although LPVs have not been studied to a great extent, they have been proposed as potential food fortification agents (Draghici et al., 2013, Foyer and Noctor, 2011, Roberts and Moreau, 2016, Sarkar and Rakshit, 2017, Banwo et al., 2021, Sarkar et al., 2022b). All of these findings point to the possibility that increasing one's consumption of LPVs might not only supply the body with the nutrients it needs for healthy growth but also offer sufficient defense against illnesses that are brought on by an inadequate diet (Sarkar et al., 2022b). Their consumption has skyrocketed as a direct result of recent efforts to raise knowledge about the nutritional advantages they offer in comparison to commonly consumed foods. These LPVs are rich in dietary fiber, vitamins, and essential minerals such as Mg, Cu, and Se (McDaniel and Jadeja, 2019, USDA, 2019, Rizvi et al., 2022). In addition to this, it is well established that they can be therapeutically useful in the treatment of chronic health disorders such as cancer and cardiovascular illnesses (Verma et al., 2018b, Gutierrez et al., 2019, Silva et al., 2021, Verma et al., 2021a, Rizvi et al., 2022). This is because these LPVs contain essential pigments including carotenoids, anthocyanins, and other phenolic compounds that can function as natural antioxidants (Leja et al., 2010, Mizgier et al., 2016, Verma et al., 2018b, Kaur et al., 2021, Gao et al., 2022, Sarkar et al., 2022b). In addition, there are several chemical components, the most notable of which is glutathione (γ-glutamyl-L-cysteinyl-glycine, abbreviated as GSH) (Noctor et al., 2012, Zhou et al., 2022). Glutathione may be found in a variety of LPVs, including those spinach and red cabbage that can fulfill their role as natural antioxidants. Other vegetables also contain glutathione (Noctor et al., 2012, Kou et al., 2019, Pradedova et al., 2019, Lai et al., 2021, Li et al., 2021, Nacca et al., 2021, Sun et al., 2022). Glutathione is the most prevalent non-protein thiol molecule and is mostly found in eukaryotic cells. It is broadly dispersed across all living species (Öztetik, 2008, Foyer and Noctor, 2011, Noctor et al., 2012). Even though the reduced form of glutathione makes up more than 90 % of the glutathione that is generally present, various other forms of glutathione may be found in microbial cells, tissues, and plasmas (Öztetik, 2008, Foyer and Noctor, 2011, Noctor et al., 2012). After glutathione is oxidized, glutathione disulfide GSSG (oxidized glutathione) is produced. This oxidized glutathione may then be reduced back to glutathione by glutathione reductase, although the process uses up NADPH in the process (Carmel-Harel and Storz, 2000, Öztetik, 2008, Mahmood et al., 2010, Foyer and Noctor, 2011, Noctor et al., 2012). In addition to GSSG, glutathione may be found in several different mixed disulfide forms, such as GS-S-CoA, GS-S-Cys, and GS-S-protein, which is produced by the process of glutathionylation (Penninckx, 2002, Foyer and Noctor, 2011, Noctor et al., 2012, Shu et al., 2016). Although it has been revealed that glutathione is involved in a great number of physiological processes and plays a variety of important functions, the primary and general roles of glutathione can be summed up in three major ways: serving as an antioxidant, boosting the immune system, and detoxifying higher eukaryotic organisms (Pastore et al., 2003; Tausz et al., 2004; Öztetik, 2008, Foyer and Noctor, 2011, Noctor et al., 2012). To begin, the powerful capacity of glutathione to donate electrons and its relatively high intracellular concentration (which may reach a millimolar level) makes it possible to keep a reducing environment within the cell (Foyer and Noctor, 2011, Noctor et al., 2012, Aquilano et al., 2014). Because of this, glutathione is an essential antioxidant that protects DNA, proteins, and other macromolecules from oxidative damage that can be caused by reactive oxygen species (ROS), for example. Second, glutathione is one of the most powerful antiviral chemicals that research has discovered, and it is also very essential to the functioning of the immune system through its contribution to the formation of white blood cells. In conclusion, to complete the detoxification process, glutathione can be conjugated to various exogenous electrophiles and xenobiotics through the action of glutathione-S-transferase (Foyer and Noctor, 2011, Noctor et al., 2012, Shu et al., 2016). As a result, glutathione is often regarded as one of the most effective, adaptable, and significant naturally-occurring defensive chemicals (Foyer and Noctor, 2011, Noctor et al., 2012, Aquilano et al., 2014). Glutathione shortage has been associated with a multitude of disease states in humans, including HIV infection, liver cirrhosis, lung disorders, inflammations of the gastrointestinal tract and pancreas, diabetes, neurodegenerative diseases, and aging (Wu et al., 2004, Öztetik, 2008, Gaucher et al., 2018, Bertoni et al., 2019). Glutathione has the potential to be utilized as an additive in the food industry as well as the cosmetics industry (Murata, 1989, Sies, 1999, Wu, 2018, Sonthalia et al., 2016). Glutathione is utilized most frequently as a chemical in the pharmaceutical industry; nevertheless, it possesses the potential to be utilized in the aforementioned other industries as well (Murata, 1989, Locigno and Castronovo, 2001, Day, 2005, Ally et al., 2021). In point of fact, the reason that glutathione has such a wide range of applications in the industry is that this chemical is usually recognized as being risk-free for consumption in the form of a nutritional supplement. On the other hand, there are several reports on an oral acute toxicity study of glutathione in mice that suggest that the lethal dosage 50 (LD_50_) was more than 5 g/kg, which indicates that glutathione is harmless. In a considerable number of clinical trials, there have been no major adverse responses recorded (Witschi et al., 1992, Kern et al., 2011, Kovacs-Nolan et al., 2014, Weschawalit et al., 2017). On the other hand, it is capable of even reversing the harmful effects that result from excessive use of other amino acids (Tateishi et al., 1977). Therefore, this is suggested because there is the possibility in research and studies for additional price reduction via the development of improved production processes of glutathione. Even though the physiological functions of glutathione in animal and human tissues, as well as plant and microbial cells, have been exhaustively investigated and explained (Penninckx and Elskens, 1993, Carmel-Harel and Storz, 2000, Penninckx, 2000, Penninckx, 2002; Tausz et al., 2004; Foyer and Noctor, 2011, Aquilano et al., 2014). There are a surprisingly small number of reviews that focus on the production of this therapeutically significant tripeptide using biotechnology. A significant portion of the findings from research on the synthesis of GST may have been patented due to the significant commercial interest in the topic. The most important studies that describe the synthesis of glutathione were published first time about thirty-three years ago (Murata, 1989, Murata, 1994, Murata and Kimura, 1990). These reviews placed a particular emphasis on the manufacture of glutathione by genetically altered *Escherichia coli* or *Saccharomyces cerevisiae*. In 1888, an ethanol extract of baker’s yeast included a substance that was later dubbed “philothion.” This substance was later confirmed to be glutathione. Following the determination of its atomic and molecular structures in 1921, it was subsequently given the name glutathione (Penninckx and Elskens 1993). After it was recognized that glutathione was present in a wide variety of living creatures, a preparative method called the solvent extraction of glutathione from diverse plant tissues was developed and utilized. The numerous analytical methods that are now being utilized for the detection of glutathione are described in several existing papers that have been published by researchers and scientists working in the concerned area. These analytical methods include spectrofluorimetry, colorimetry, magnetic resonance imaging, high-performance liquid chromatography (HPLC), surface-enhanced Raman scattering, electrochemical methods, and mass spectrometry (Mills et al., 1997, McDermott et al., 2011, Zachariadis and Rosenberg, 2013, Zhao et al., 2014, Ni et al., 2015, Yan et al., 2016, Ngamchuea et al., 2017a, Ngamchuea et al., 2017b, AlFaris et al., 2020). Even if the majority of these methods are sensitive and precise, each one of them still has some limitations, such as the fact that it takes a lot of time, it requires expensive apparatus, and it has a convoluted procedure for carrying it out. As a result, there is a significant need for analytical procedures that are both quick and accurate, in addition to being cost-effective (Khan et al., 2016, Wabaidur et al., 2016, Siddiqui et al., 2017, Bavatharani et al., 2020, Lakshmi et al., 2020). The usage of fluorescent probes that are built on a range of fluorescence sensing platforms and that provide the benefits of high sensitivity, cheap cost, and ease of use has become increasingly common (Wabaidur et al., 2018; AlFaris et al., 2020). Due to the growing interest among consumers to improve their nutrition and combat a variety of chronic illnesses, researchers have focused a particular emphasis on the existence of antioxidants, particularly glutathione, in plants.

As a preparative method, solvent extraction of glutathione from a variety of plant tissues became popular once it was shown that glutathione is present in a significant number of living species. However, because there was a shortage of raw materials and the intracellular concentration of glutathione was not very high, the finished product was somewhat pricey, which hampered its ability to be put to practical use. As a result, the purpose of the study was to successfully extract and identify glutathione from plant leaves such as spinach and red cabbage using a variety of approaches. In addition, the goal of this study was to determine the amount of glutathione that could be used in meat patties and to study some of their physical and chemical properties for preservation susceptibility in meat patties.

## 2 Materials and methods

### 2.1 Plant materials

The leaves of spinach and red cabbage that were used were obtained by making purchases at the local marketplaces in the city of Basrah. They were dried at a temperature of 40 °C for a period of time that did not exceed 12–48 ± 2 h. After that, they were crushed up using an electric grinder, and the material that had been dried was stored in the freezer so that its quality would be maintained until it was used for laboratory experiments.

### 2.2 Extraction and measurement of glutathione from plant leaves

The method developed by Xiong et al. (2009), with some modifications, was utilized for the extraction and measurement of glutathione found in the leaves of spinach and red cabbage plants. Powdered plant samples weighing a total of 10 gm were added to each of the three extraction solutions, which consisted of either 100% water, 100% ethanol, or a mixture of 30% ethanol and 70% water by volume (v/v). Each solution was given a total volume of 200 mL. After allowing the combination from each extraction solution to be thoroughly stirred for 24 h using a plate equipped with a magnetic stirrer, filter the solution using Whatman filter paper No. 1. At a temperature of 40 °C, the filtrate was collected using a rotating vacuum evaporator, and it was then concentrated. After that, the extract was lyophilized to remove moisture, put in opaque containers with secure lids, and stored at a temperature of −18 ± 1 °C until it was ready for use. In addition, to separate glutathione from plant extracts, an ultrafiltration apparatus was utilized as described by Al-Hilfi and Almosawi (2016), and the mixture was passed through membranes having pore sizes of 30, 10, and 5 kDa respectively. After the extracts were concentrated using a rotary evaporator at a temperature of 40 °C, they were lyophilized by utilizing freeze-drying, and then they were kept in the freezer until they were needed. Nitrogen gas was injected at a pressure of 5 bar.

### 2.3 Determination of antioxidant efficacy of plant leaf extracts using DPPH

The methodology developed by Xu et al. (2010) was applied with minor adjustments made to the concentrations to determine the level of antioxidant activity possessed by the extracted glutathione. To produce a solution of DPPH with a concentration of 0.01%, methanol was utilized. The procedure consisted for preparing concentrations of the extracted glutathione that ranged from 10 to 50 mg/mL. This was accomplished by combining 1 mL of each concentration with 0.01% DPPH and then allowing the mixture to remain in the dark for 30 min. After that, the absorbance was determined by using a wavelength of 517 nm, the synthetic antioxidant butylated hydroxytoluene (BHT) was utilized in the same concentrations for the comparison, and the control sample was prepared in the same manner as the test sample, with the exception that methanol was added in place of the sample. The following equation was used to determine the proportion of free radicals that were successfully recorded:

$$Effectiveness=\frac{\;For\;extract\;absorbance\;reading\;-\;control\;for\;absorbance\;reading}{\;90\%\;Absorbance\;reading\;for\;the\;control\;sample\;Absorbance\;reading\;for\;methanol}\times 10.$$

### 2.4 Identification and characterization of glutathione from plant leaf extract

#### 2.4.1 Thin-layer chromatography (TLC) study of plant leaf extract for glutathione concentrations

The methodology developed by Ma et al. (2010) was utilized to qualitatively detect glutathione in the extracts obtained from spinach leaves and red cabbage leaves. As a fixed phase, we employed silica sheets that were 20 × 20 cm in dimensions. The mobile phase was made up of water, glacial acetic acid, and butanol in the following ratios: 3:2:1. The extract containing glutathione was injected at a distance of 2 cm from the bottom edge of the plate. Once the solvent had reached 2 cm before the end of the plate, the plates were patched, and after allowing them to dry for 5 min, they were sprayed with a solution that contained 1 % ninhydrin solution. A drying process was performed on the plate in an oven set at a temperature of 40 °C until the areas were visible. The value of the retention factor (R*_f_*) was determined by applying the following equation to the calculation:

$$R_{f}=\frac{\;Distance\;Travelled\;by\;the\;Sample}{\;Distance\;Travelled\;by\;the\;Solvent}.$$

where

R*_f_* is a retention factor

#### 2.4.2 Ultraviolet–visible (UV–Vis) spectral analysis

Ultraviolet–visible (UV–Vis) spectral analyses were carried out in the manner suggested by Al-Haji (2006). To find the glutathione wavelength with the maximum absorbance, spectrophotometry was employed in conjunction with a spectrum of wavelengths spanning from 200 to 400 nm. This allowed for the most accurate measurement of the optimal glutathione wavelength.

#### 2.4.3 Fourier transform infrared (FTIR) spectroscopy analysis

Fourier transform infrared (FTIR) spectroscopy studies were performed as described by Sharma (2009) to identify and characterize glutathione, and peptides were recorded from 4000 to 400 cm^−1^ using a spectrometer (Jasco FTIR-4200, Japan) with KBr serving as the sample matrix. To produce meat patties, samples were initially mixed with KBr before being pressed to form meat patties. A total of ten scans were taken, each with a resolution of cm^−1^.

#### 2.4.4 High-performance liquid chromatography (HPLC) analysis

Following the methodology laid out by Dahl-Lassen and colleagues, HPLC (SYKAM, Korea) was utilized to determine the quantity of different amino acids present in glutathione samples (Dahl-Lassen et al., 2018). The volume-to-volume ratio of the mobile phase’s components was as follows: 60% acetonitrile, 20% methanol, and 20% formic acid. After dissolving 3 g of each sample in 25 mL of 6% hydrochloric acid (HCl) at a temperature of 150 °C for 3 h, the samples were then allowed to dry. Following the addition of 5 mL of sodium citrate (Na_2_C_6_H_5_O_7_) to the samples that had been dried at a pH of 2.2, the samples were filtered using a polypropylene filter. After that, 1 mL was taken out of the extracted form, and 200 μL of ortho-phthalaldehyde (OPA) or paraldehyde with a concentration of 5% was added to it. Two minutes were spent shaking the mixture well. The volume of the injection was 100 µL, and the flow rate was 0.8 mL/min. The ZORBAX Eclipse-AAA column that was utilized had an internal diameter of 3.5 µm, 4.6 × 150 mm, and a temperature of 25 °C. Fluorescence was used as the technique for determining the presence of glutathione (Ex = 445 nm, Em = 465 nm). All of the data were reported in terms of mg/100 g of dry weight (DW), and their values were presented in terms of the mean as well as the standard deviation of three separate observations.

### 2.5 Test of the physical and chemical properties of glutathione for preservation susceptibility in meat patties

**Fig. 1:**
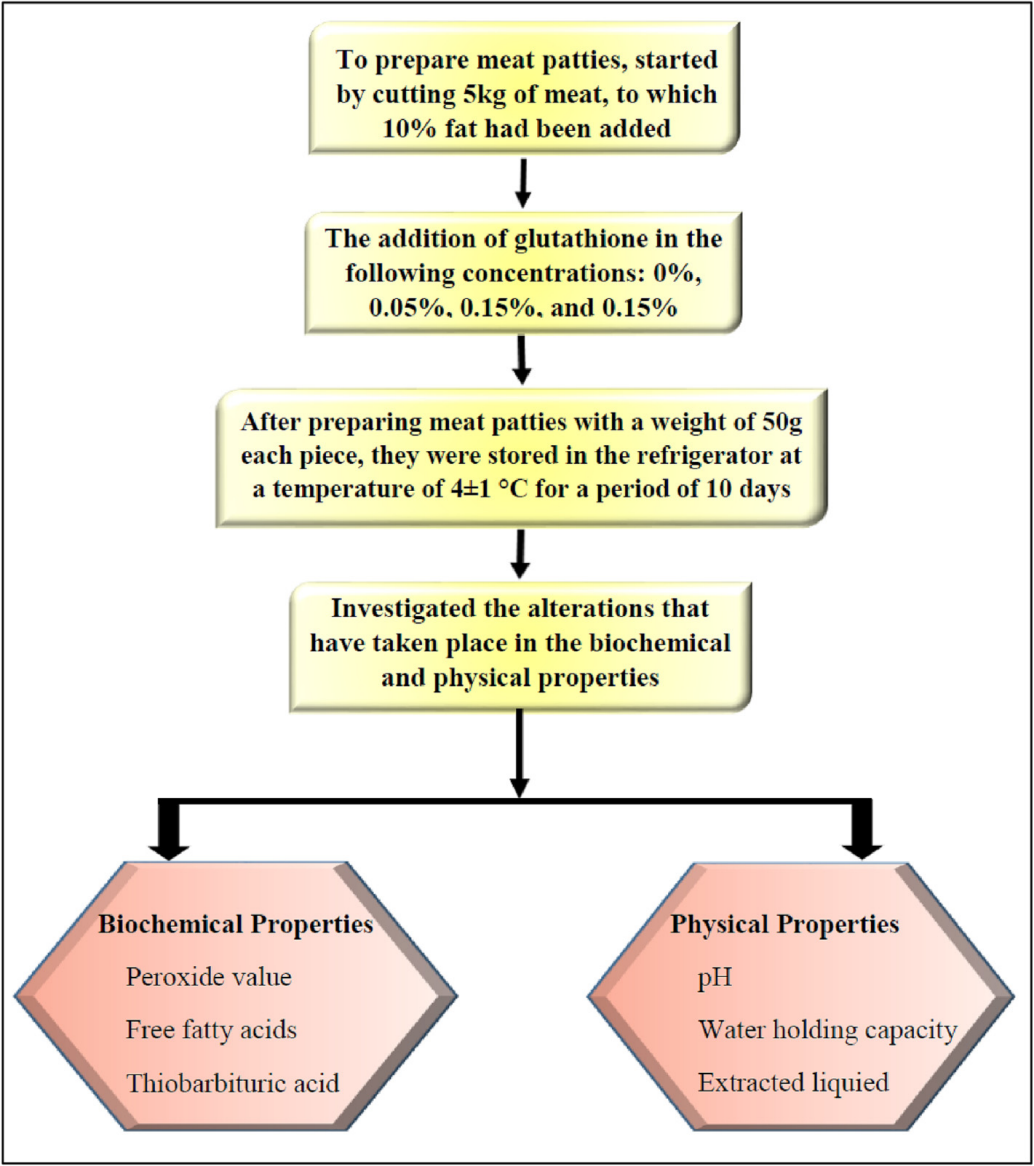
Diagrammatic representation of the steps involved in the preparation of meat patties.

The several steps involved in the manufacturing process of meat patties are illustrated in Fig. 1. The preparation of meat patties began with the mincing of 5 kg of meat using an electric machine with a whole diameter of 3 mm. Following this step, 10 % of the fat was added to the meat, and the mixture was minced once more to ensure that the fat was evenly distributed throughout the meat. After that, the meat patties were divided into four treatments, designated T1, T2, T3, and T4. T1 represented the control sample, which did not have any glutathione added to it. T2, T3, and T4 represented samples to which glutathione was added at weight-per-weight (w/w) concentrations of 0.05:100, 0.1:100, and 0.15:100, respectively. All of the meat pattie treatment samples were refrigerated for 10 days at 4 ± 1 °C, and then the changes in pH, water-holding capacity (WHC), extracted liquid volume, peroxide value, percentage of free fatty acids (FFAs), and thiobarbituric acid (TBA) value were evaluated, which were associated with the analysis of the changes in physical and biochemical properties.

#### 2.5.1 Physical property tests

Physical property tests were carried out according to the method described by Mendenha (1989). The pH was determined after mixing 10 g of the sample with 20 ml of distilled water, letting the mixture sit for five minutes, and then measuring the pH of the meat patties. The volume of the liquid that was drained from the meat patties had estimated to be the WHC.

#### 2.5.2 Chemical property tests

The testing of the chemical properties was carried out following the standard procedures that were already described in the available literature. The method described by Pearson (1976) was utilized to determine the peroxide value (PV) of meat patties. The method proposed by Al-Tai and Al-Mousawi (1992) was utilized to estimate the percentage of FFAs present in meat patties. Subsequently, the method proposed by Soltanizadeh and Ghiasi-Esfahani (2015) was utilized to determine the value of TBA.

### 2.6 Statistical analysis

Statistical analyzes for data were designed using a completely randomized design (CRD). All measurements were performed in triplicate for each sample, and at least three independent analyses of each sample were reported. For the analysis of variance (ANOVA) on the experimental data, all statistical analyses were performed using SPSS software version 13.0, 2009. For the ANOVA, the least significant difference (LSD) range was utilized to evaluate the significance of the differences, which was attributed to the 0.05 level.

## 3 Results

### 3.1 Extraction and measurement of glutathione from plant leaves

**Fig. 2:**
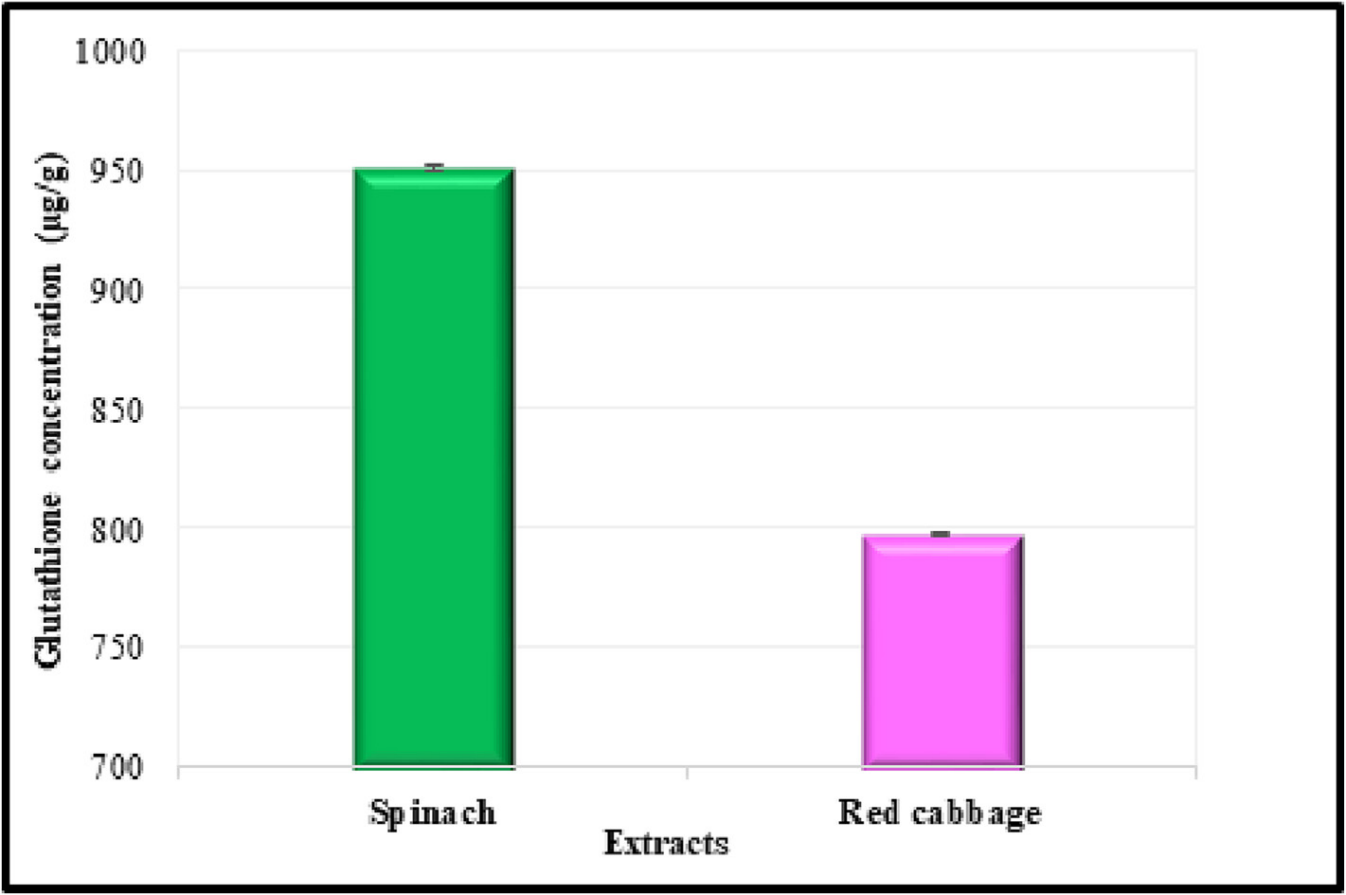
A quantitative depiction of the concentration of glutathione found in spinach and red cabbage plant extracts.

The concentration of glutathione derived from the plant extracts of spinach and red cabbage is depicted in the diagram (Fig. 2). The results showed that the average concentration of glutathione present in the spinach leaf samples extracted using ethanol and water at a ratio of 30:70% (v/v) was 951 µg/g. According to the findings, the glutathione content in spinach leaves was much higher than that of red cabbage leaves, coming in at 797.5 µg/g.

### 3.2 Antioxidant efficacy of plant leaf extracts using DPPH

**Fig. 3:**
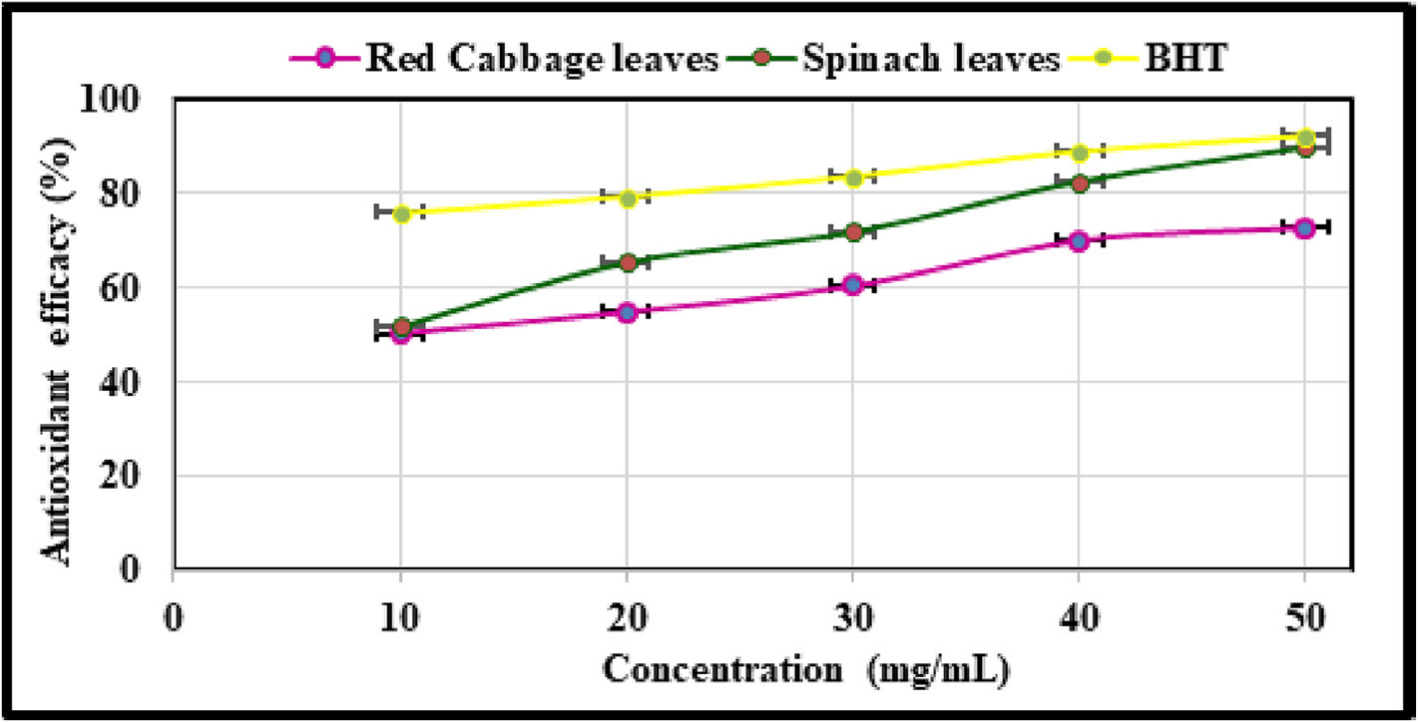
Graphical representation of the antioxidant capacity of plant leaf extracts determined using DPPH.

The DPPH antioxidant activity of glutathione derived from spinach leaves and red cabbage leaves is depicted in Fig. 3. The DPPH antioxidant activity reported was much lower than that of BHT. The findings have also been illustrated in Fig. 3, which demonstrates that the antioxidant activity rises as the concentration of glutathione rises from 10 mg/mL to 50 mg/mL. At a concentration of 10 mg/mL, the antioxidant activity reaches a maximum of 50.65%, while at a concentration of 50 mg/mL, it reaches a maximum of 50.23%. At a concentration of 50 mg/mL, the antioxidant activity of spinach leaf extract was comparable to the antioxidant activity of BHT at the same concentration, which was 92.18%.

### 3.3 Identification and characterization of glutathione from plant leaf extract

#### 3.3.1 TLC study of plant leaf extract for glutathione concentrations

**Fig. 4:**
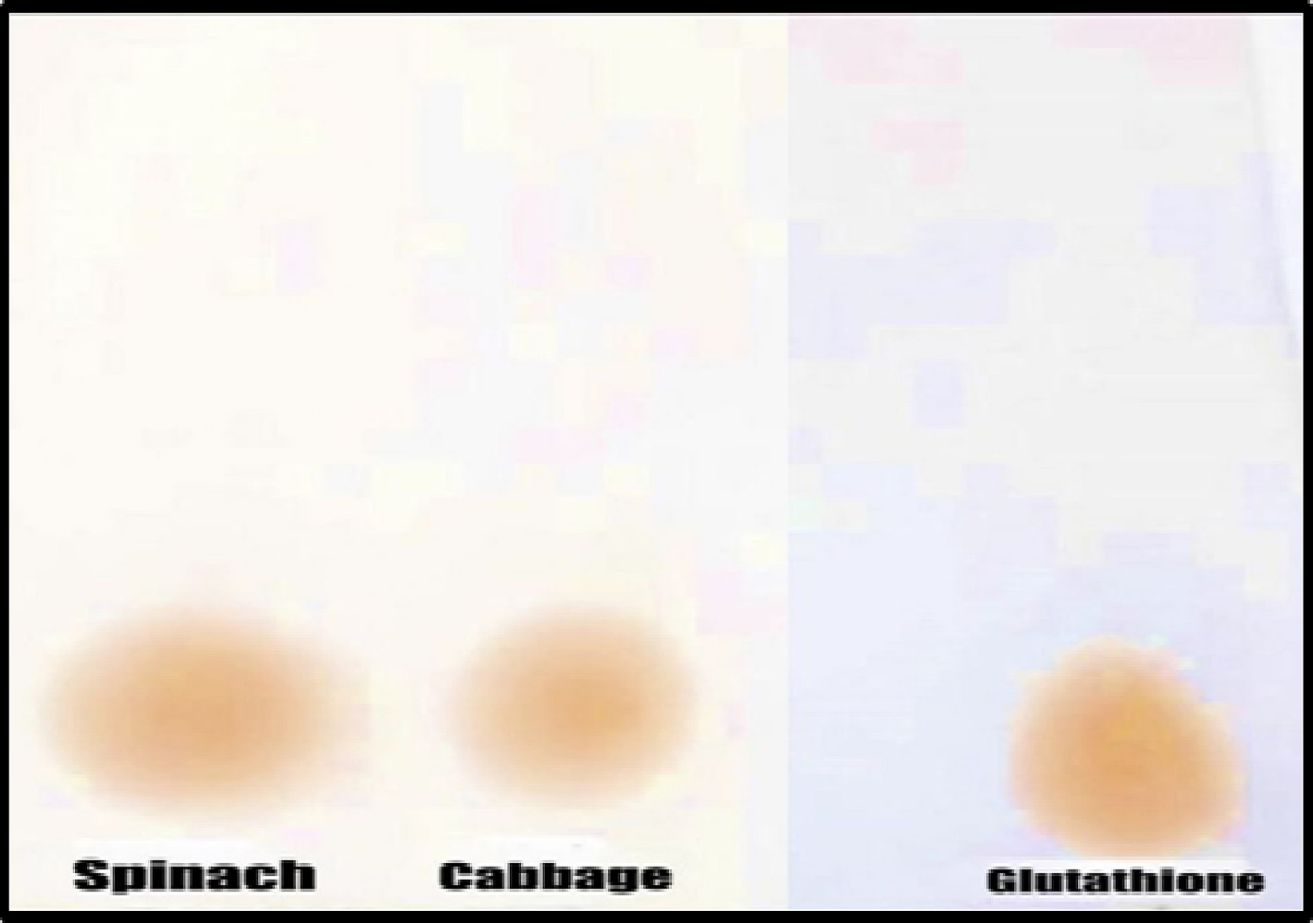
A graphical illustration of the outcomes of the thin-layer chromatography performed on plant leaf extracts.

The results of a TLC glutathione analysis have been presented in Fig. 4 and Table 1. This analysis was performed to establish the level of purity of glutathione that was extracted from spinach leaves and red cabbage leaves. Glutathione spots were observed for plant extracts. The R*_f_* value was the same as the R*_f_* value for the extract of spinach leaves, and the R*_f_* value for the extract of red cabbage was 0.164. The value of the R*_f_* was very close to reaching 0.176 for the standard glutathione, and this value was identical to the R*_f_* value for the extract of spinach leaves.

**Table 1:** Flow rate for glutathione that was extracted from the leaves of the plant and glutathione that was used as standard.

Source of Glutathione	Flow rate
Red Cabbage leaves	0.164
Spinach leaves	0.176
Standard Glutathione	0.176

#### 3.3.2 UV–Vis spectral analysis

**Fig. 5:**
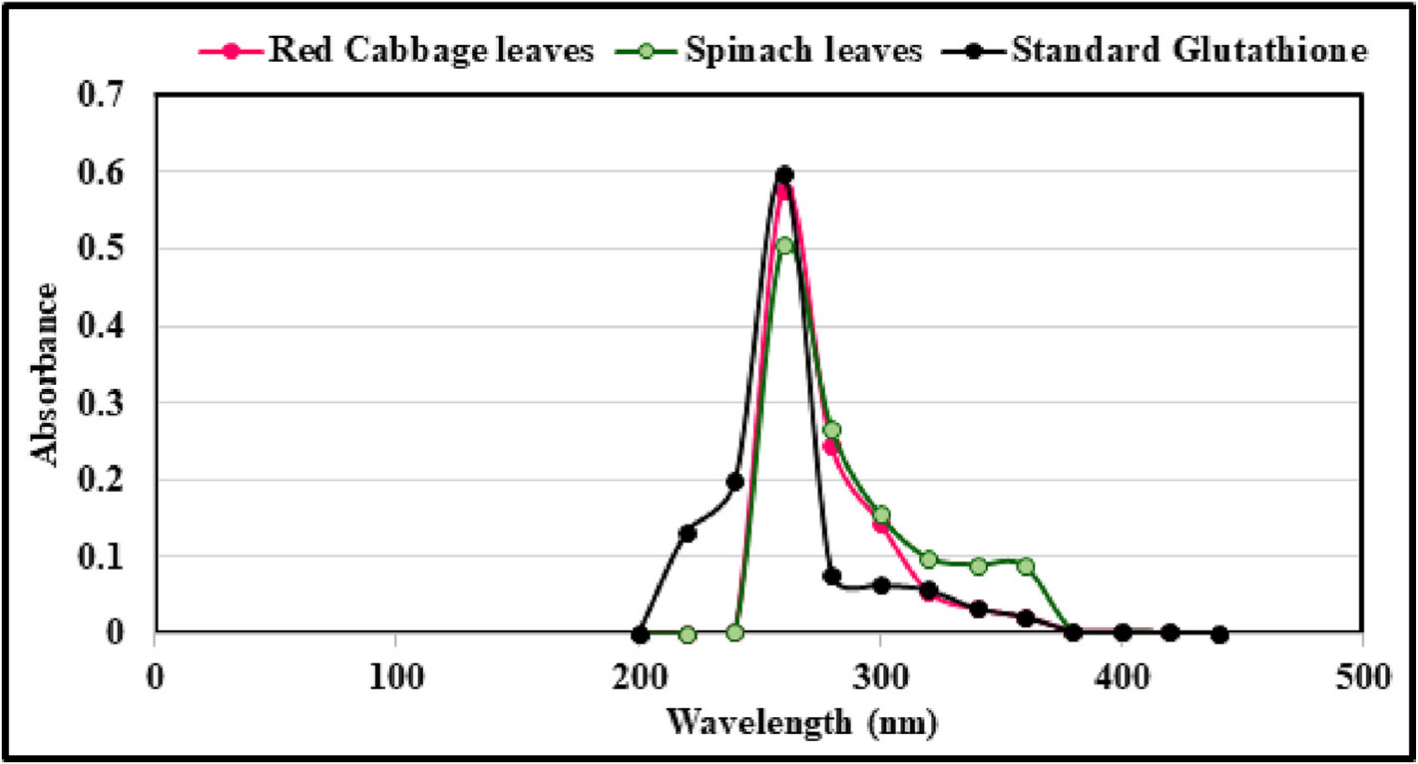
Optimum wavelength of glutathione for spinach leaves, red cabbage leaves and standard glutathione.

Results presented in Fig. 5, which were acquired from the current investigation, the optimal wavelength of glutathione extracted from spinach leaves, red cabbage leaves, and standard glutathione was detected at a wavelength of 260 nm, with varying absorbances for the various plants that were investigated. In our investigation on the optimum wavelength at which to measure glutathione, the findings came to some conclusions. In addition, the results demonstrated that the peptides measure the absorbance at invisible wavelengths (ultraviolet), which occurred in the region of 200–300 nm.

#### 3.3.3 FTIR spectroscopy analysis

**Fig. 6:**
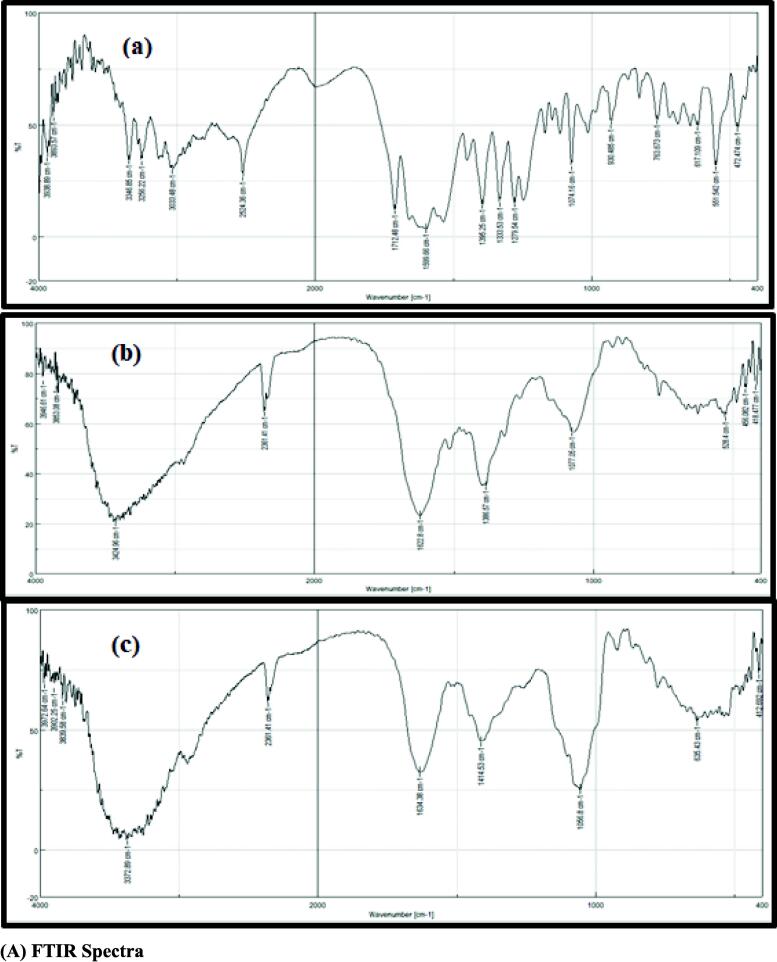

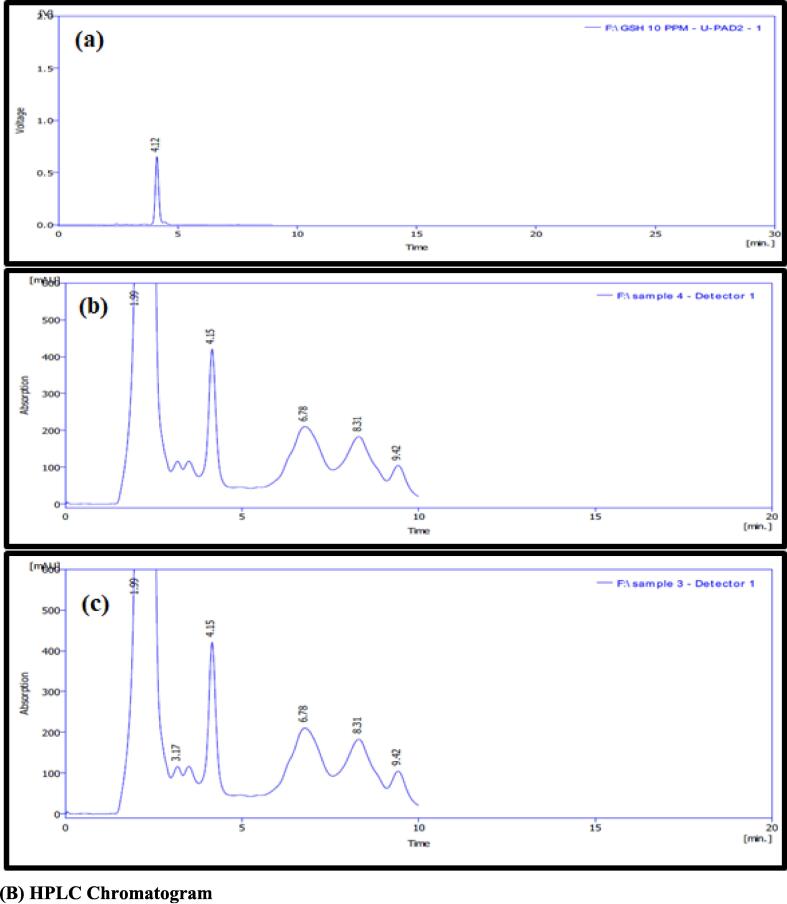
Characterization of glutathione using different analytical instruments. (a) Standard Glutathione, (b) Glutathione Spinach leaves, and (c) Glutathione Red Cabbage leaves.

The FTIR spectra of a pure peptide fraction isolated from spinach leaves, red cabbage leaves, and standard glutathione are presented in Fig. 6(A). The effective group–SH can be shown in Fig. 6(A) at the wave number 2361.41 cm^−1^ for the extracts that were employed, and this wave number was very near to the wave number of the same group for glutathione standard, which was 2524.36 cm^−1^. This seemingly little change could be because this group is only barely detectable by the infrared spectrometer, unless. The material was 100% pure; thus, the existence of any contaminants modified the appearance of this band, particularly the extracts, as well as the frequency location; nonetheless, the frequency shown in the table for extracts is still within the range that this group covers.

#### 3.3.4 HPLC analysis

As can be seen in Fig. 6(B), the present analysis demonstrated that the peaks of glutathione that were extracted from the spinach and red cabbage leaves had a retention duration of 4.15 min. The findings indicated that the samples of glutathione extracted from spinach leaves and red cabbage leaves that were analyzed by HPLC had identical peaks with identical retention times, even though the concentrations of glutathione in each of the samples varied. The average amount of time that glutathione was retained was 4.12 min. According to the findings, the retention times of the extracted glutathione were quite similar to those of the reference glutathione. The examination with HPLC revealed the glutathione concentrations in the extracts, which reached 58.9 ppm and 44.8 ppm, respectively, in the spinach and red cabbage extracts. According to the findings, the concentrations of the three amino acids that are necessary for the production of glutathione—namely cysteine, glutamic acid, and glycine—in glutathione found in spinach leaves were 53.6, 57.9, and 44.5 ppm, respectively. These amino acids were all involved in the production of glutathione. In comparison, the concentration of cysteine was 42.9 ppm in glutathione-rich red cabbage leaves, while the concentration of glutamic acid was 35.9 ppm and the concentration of glycine was 30.2 ppm.

### 3.4 Physical and chemical properties of glutathione-incorporated meat patties

#### 3.4.1 pH

**Fig. 7:**
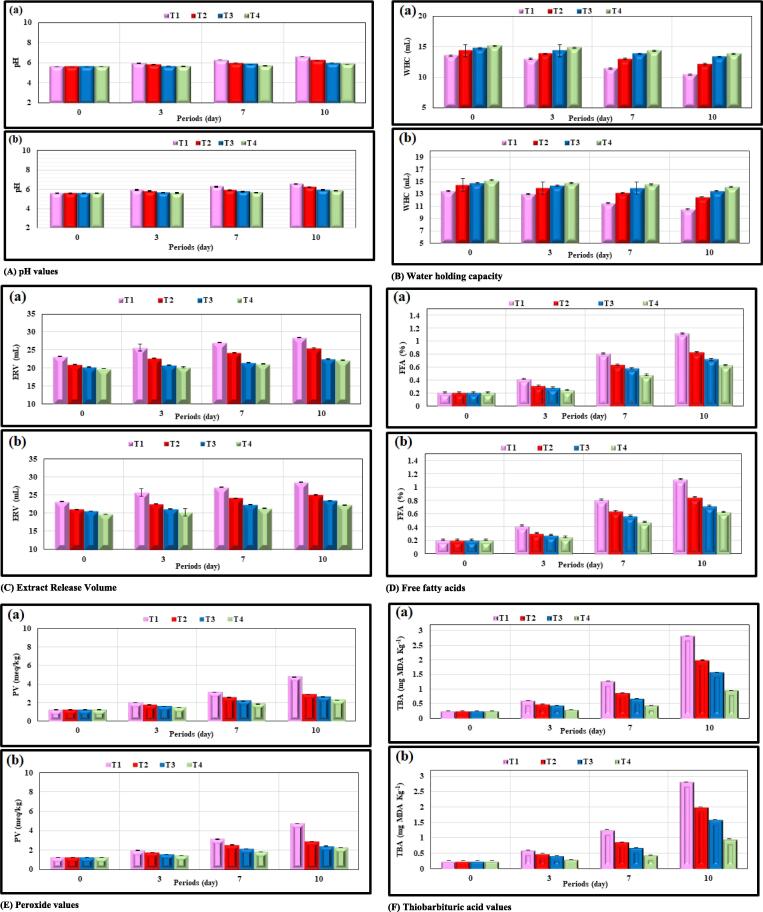
Effect of extracted glutathione on various parameters of meat tablets. (a) Glutathione Spinach leaves, and (b) Glutathione Red Cabbage leaves. [*Note:* T1: Control; T2: 0.05:100 (w/w); T3: 0.1:100 (w/w); T4: 0.15:100 (w/w)].

The data presented in Fig. 7(A) illustrates the impact that the addition of glutathione had on the pH levels of meat patties that had been kept in cold storage at a temperature of 4 ± 1 °C. Furthermore, it was found that the concentration that was utilized had an impact on the pH of the solution. The pH value dropped as the concentration that was used was increased from 0.05:100 (w/w) to 0.15:100 (w/w), which was the range that was utilized. The presence of peptides that inhibit the growth of bacteria that produce proteolytic enzymes in the meat is the cause for this phenomenon. It rises as the length of time spent in cold storage continues, but the rate of rise was lower in the meat patty samples that had glutathione added to them in comparison to the treatment that served as the control. After being kept in the refrigerator for ten days, the value increased to 6.25, 5.97, and 5.86 correspondingly at concentrations of 0.05:100 (w/w), 0.1:100 (w/w), and 0.15:100 (w/w), respectively.

#### 3.4.2 Water holding capacity

As can be seen in Fig. 7(B), the meat patty samples that had glutathione added to them had a higher WHC than the control sample. It was 14.3 mL in samples that included glutathione extracted from spinach leaves and 14.5 mL in samples that contained glutathione extracted from cabbage leaves, both at a concentration of 0.05:100 (w/w). The WHC of the control treatment declined during the storage period, moving from 13.5 mL at the beginning of the storage period to 10.5 mL on the tenth day of storage. At the end of the storage period, the WHC of the meat patties that had been treated with spinach leaf glutathione (form A) had fallen to a concentration of 12.2 mL with a weight-to-weight ratio of 0.05:100. After 10 days of storage, the WHC of the red cabbage glutathione fell to 12.5, 13.5, and 14.2 mL at the concentrations of 0.05:100 (w/w), 0.1:100 (w/w), and 0.15:100 (w/w), respectively. This was the case at all three concentrations depicted in Fig. 7(B). Fig. 7(B) reveals that increasing the concentration of glutathione extracted from the leaves of spinach and red cabbage led to an increase in the WHC of the meat patties.

#### 3.4.3 Extract release volume

The values of the volume of the extracted liquid in the frozen-stored meat patties treated with glutathione from spinach and red cabbage leaves were significantly affected by the added concentration, as a decrease in the volume of the extracted liquid can be seen in Fig. 7(C), which demonstrates the results of the experiment. The volume of the liquid that was extracted by increasing the additional concentrations of glutathione reduced from 0.05:100 (w/w) to 0.15:100 (w/w) as the concentration rose. The glutathione extracted from cabbage leaves was applied at a concentration of 0.15:100 (w/w) to a volume that was 19.8 mL, 20 mL, 21.1 mL, and 22.2 mL throughout the periods of 0 days, 3 days, 7 days, and 10 days, respectively Fig. 7(C). At the beginning of the storage stage, the extracted liquid had a concentration of 0.15:100 (w/w) in 19.7 mL, and by the end of the storage stage, it reached 22.2 mL. Both of these values were lower in comparison to the control treatment, as we observe from Fig. 7(C), which shows that the volume of the extracted liquid gets rid of the increased concentration of glutathione added.

#### 3.4.4 Free fatty acids

The impact that the addition of glutathione had on the total amount of FFAs found in frozen meat patties based on the results can be seen in Fig. 7(D). The results showed that the value of FFAs for meat patties treated with extracted glutathione was 0.21% at the beginning of the storage stage. As the amount of glutathione that was added to the beef patties increased in concentration, the levels of FFAs that were measured in the patties dropped. For instance, the value of FFAs in meat patties treated with glutathione derived from spinach leaves (Fig. 7(D)) decreased from 0.83% to 0.63% at a concentration of 0.05:100 (w/w) and then increased to 0.15:100 (w/w) at the end of the stage. This occurred at the end of the stage when the glutathione concentration was increased to 0.15:100 (w/w). The same result had been achieved by using glutathione that had been isolated from red cabbage leaves (Fig. 7(D)). Throughout the 10-day storage period, the results demonstrated a rise in the FFAs values of meat patties treated with glutathione extracted; however, in comparison to the treatment that served as the control, this increase was significantly lower.

#### 3.4.5 Peroxide value

Fig. 7(E) depicts the peroxide values of the minced meat patties that were supplemented with glutathione extracted from the leaves of spinach and red cabbage leaves and stored by refrigeration at a temperature of 4 ± 1 °C for a period of 3, 7, and 10 days. Peroxide values for all treatments at the beginning of storage was 1.23 mEq/kg, and the peroxide values in meat patties treated with glutathione in spinach leaves (Fig. 7(E)) amounted to 2.25 mEq/kg on the tenth day of storage at a concentration of 0.15:100 (w/w) (Fig. 7(E)). The control treatment had a concentration of 0.15:100 (w/w), which resulted in a value of 2.24 mEq/kg at the end of the storage phase.

#### 3.4.6 Thiobarbituric acid

Fig. 7(F) illustrates the effect that adding glutathione had on the levels of TBA that were present in meat patties that had been chilled for an extended period. At the end of the storage phase, with glutathione produced from spinach leaves and red cabbage leaves, forms (A) and (B), 0.965 mg malonaldehyde/kg were produced at a concentration of 0.15:100 (w/w). This was less than the control treatment, which reached 2.814 mg malonaldehyde/kg at the end of the storage phase, which is 10 days.

## 4 Discussion

### 4.1 Extraction and measurement of glutathione from plant leaves

When it comes to the recovery of phytochemicals, the extraction process is also highly important. To obtain optimal extraction efficiency, it is necessary to take into consideration both the nature of the plant materials and the nature of the bioactive components (Tsao and Deng, 2004, Chávez-González et al., 2020, Verma and Srivastav, 2020b, Tripathy et al., 2021, Tripathy et al., 2022). The metabolite glutathione has been identified as having several functions, and it is found naturally in a variety of plant species (Alscher, 1989, Noctor et al., 2012, Pradedova et al., 2019, Nacca et al., 2021, Sun et al., 2022). It is a considerable reservoir of nonprotein-reduced sulfur and serves important activities not just in plants but also in animals and humans (Ngamchuea et al., 2017a). As a result, the extraction of this naturally occurring metabolite with several functions as well as the determination of its concentration has become a significant area of focus. In this regard, the technique for the preparation of samples to be used in the research of the glutathione content in plant material consists of at least three steps: (1) initial sample preparation; (2) extraction or leaching of soluble components of the substance being studied with appropriate solvents; and (3) analytical enrichment while simultaneously removing interferences through the use of methods (Romanik et al., 2007). The first two are always used, although the third is common but not necessary to be used. This was addressed in the lines that came before this one. It was observed that the levels of glutathione in each of the samples were noticeably different from one another. It is possible that the differences in glutathione concentration between plant species were caused by factors such as the variety that was used, the ripening period, the geographical origin, the growing season, the post-harvest storage conditions such as temperature and storage period, as well as the treatment procedures that were performed on the plants (Łata et al., 2005). Previous research conducted by Nacca et al. (2021) revealed that the concentration of glutathione in one of the varieties of red cabbage leaves was 895 µg/g. The glutathione concentration that was observed in the current investigations was in agreement with the findings of the previous research (Nacca et al., 2021).

### 4.2 Antioxidant efficacy of plant leaf extracts using DPPH

Antioxidants regulate and prevent the oxidative damage that occurs in foods by delaying or suppressing the oxidation that is induced by ROS. This eventually increases the shelf-life and quality of the foods in consideration (Banwo et al., 2021, Verma and Thakur, 2021). The results of many experiments demonstrate the possibility that ROS and free radicals are involved in a wide variety of disorders (Scartezzini and Speroni, 2000). Plants are an essential source of production of a large number of antioxidants, which are used to reduce the effects of oxidative stress (Scartezzini and Speroni, 2000, Banwo et al., 2021). Based on their mechanisms and their biological characteristics, these unique antioxidants have the potential to serve as a source for the development of novel molecules that exhibit antioxidant activity (Scartezzini and Speroni, 2000, Verma and Thakur, 2021). Xu et al. (2010) demonstrated that the effect was increased by increasing the concentration of glutathione, and they reached the highest concentration of 79.37%. The results of this study agreed with the findings of Xu et al. (2010), which showed that the effect was increased by increasing the concentration of glutathione. H-atom attached to the DPPH radical was the factor that contributed to its stability (Gupta, 2015). In addition, the presence of a thiol group is not the only factor that determines the antioxidant property; the presence of other functional groups can also improve the antioxidant activity. Furthermore, the spatial arrangement of glutathione can impact antioxidant activity by shifting the major glutathione connection from a peptide-α bond to a peptide-γ bond. As a result, glutathione compounds can be employed to prevent oxidative stress from occurring (Kairane et al., 2012).

### 4.3 Identification and characterization of glutathione from plant leaf extract

#### 4.3.1 TLC study of plant leaf extract for glutathione concentrations

One of the efficient separation methods, TLC has several benefits. These benefits include a high sample purification efficiency, a large sample load capacity, a fast analysis time, and easy extraction stages (Kumar et al., 2013, Zhou et al., 2020, Varshney et al., 2023). TLC can potentially facilitate the purification and extraction of various chemicals within a certain polarity range in the complex sample matrix since it differentiates compounds based on the polarity of the molecules that make them up (Kumar et al., 2013, Schlemmer et al., 2020, Varshney et al., 2023). Therefore, TLC analysis for the identification of particular compounds is carried out to make the most of the number of positive tests done on plant leaf extracts. The findings of the present study were comparable to those obtained by Landino et al. (2010) in their investigation into the significance of the flow rate of glutathione, which was determined to be 0.15. As various scientists and researchers demonstrated that TLC is a stable, environmentally friendly, and cost-efficient chromatographic separation technology (Kumar et al., 2013, Zhou et al., 2020, Varshney et al., 2023). This is because TLC plates have a high number of distinct layers, which are known as theoretical plates (Zhou et al., 2020). Therefore, the findings from TLC demonstrate that TLC is a method that is well adapted to and efficient for detecting and characterizing glutathione from leaf extracts of plants such as spinach and red cabbage. According to the findings of this TLC analysis in the present investigation, it was used in the pretreatment of plant leaf extract samples, such as spinach and red cabbage, and is highly recommended to resolve matrix interferences in the process of recognizing and characterizing glutathione.

#### 4.3.2 UV–Vis spectral analysis

The quantification of organic compounds employing UV–Vis spectrophotometry has been an established practice for a long time. This technique works by measuring the amount of light absorbed in the UV–Vis spectrums (Dhivya and Kalaichelvi, 2017, Patle et al., 2020). The vast majority of phytochemicals, including glutathione, polyphenols, and carotenoid pigments, include covalently bonded double bonds or aromatic systems that can absorb light in this area. Quantification of light absorbance is no longer the sole purpose served by the application of UV–Vis detectors in separation technologies, notably the combination of DAD and HPLC (He et al., 1997, Tsao and Deng, 2004, Patle et al., 2020). In the process of identifying phytochemicals, HPLC–DAD has been an essential tool, notably in the case of glutathione, polyphenols, and carotenoid pigments. It is possible to extract the spectrum information of recognized standards by the use of HPLC–DAD, and then preserve that information as a library database (He et al., 1997, Tsao and Deng, 2004). It can scan, store, and subsequently, retrieve the UV–Vis spectral data of all eluting peaks of a sample to do a comparison with the data from the library. The extremely identification of phytochemicals including glutathione can be accomplished by finding a match between the UV–Vis spectrum and the retention duration of the compound. Additionally, DAD's capabilities as a detector include the ability to concurrently detect and record chromatograms at a variety of wavelengths. This feature substantially improves the extraction system’s performance, particularly in situations where many groups of phytochemicals are co-existing inside a single sample. According to some reports, when the appropriate wavelengths are selected, such as when there is maximum absorption, it is possible to detect all classes of phytochemicals with the highest possible sensitivity (Tsao and Deng, 2004). Quantification of an unresolved or poorly resolved peak is achievable if the detection wavelength is chosen appropriately, which can also make it possible to quantify the peak. In addition to that, DAD may be utilized to investigate the purity of a peak. The conclusions of the findings were far different from those that were found in the study of Xu et al. (2010), which contributed to some interesting findings. This was because it achieved 216 nm when measured against glutathione isolated from corn germ, which has the same optimum wavelength as conventional glutathione (Xu et al., 2010). In addition, the outcomes of our current research appeared to be comparable to those of Turkey et al. (2019). These researchers noticed through their investigations that the optimal wavelength of standard glutathione was 288 nm. Since the glutathione solution was colorless when it was measured, they concluded that the wavelength was within the acceptable range (Turkey et al., 2019).

#### 4.3.3 FTIR spectroscopy analysis

The findings were somewhat consistent with what the group of scientists and researchers reported in their investigations (Dimitrovo et al., 2010; Bertoui et al., 2019; Pourmbarak Mahnaie and Mahmoudi, 2020). As shown by Kiroula et al. (2016) in their study to identify glutathione utilizing infrared spectroscopy, the researchers noticed that the sulfhydryl (—SH) group of pure glutathione did not appear due to the weak appearance of this band in the infrared spectrometer. This conclusion was reached as a result of the fact that the sulfhydryl (SH) group of pure glutathione did not appear. Because of the FTIR data, it is reasonable to draw the conclusion that FTIR is a method that is well adapted to and efficient for recognizing and characterizing glutathione from the leaves of plant foods such as spinach and red cabbage. The current FTIR findings suggest that FTIR spectroscopy may be utilized to detect and characterize glutathione from plant leaf extracts such as spinach and red cabbage. This is supported by the fact that FTIR spectroscopy was employed.

#### 4.3.4 HPLC analysis

HPLC is an analytical method that has a high degree of adaptability in both the mobile and stationary phases. Furthermore, the elution method may be varied based on the analysis (Tsao and Deng, 2004, Varshney et al., 2023). Additionally, it can separate the compounds according to the interactions that they have with the column and the solvent phase (Varshney et al., 2023). A phytochemical examination of plant leaf extracts can be carried out with the use of HPLC, which helps detect and separate organic and inorganic solutes from the sample. However, conventional chromatographic techniques (PC, TLC, and CC) in general lack the sensitivity and resolution that are often required for trace amounts of phytochemicals (Tsao and Deng, 2004). Moreover, there is a growing need to know the photochemical profiles of different plants and among different varieties of the same plant (Varshney et al., 2023). In the last 10 years, the vast majority of researchers and scientists have concluded that HPLC is possibly the most well-known and dependable method among all chromatographic separation techniques for the separation of phytochemicals (Varshney et al., 2023). The adaptability of HPLC is helped by the fact that it can use a variety of separation modes and types of detection systems. One of these detection methods is the diode array detector (DAD), which can be linked with a mass spectrometer (MS) (Tsao and Deng, 2004). The results of the current study were found to be greater than those reported by Mills et al. (1997) in a study that was carried out on various vegetable samples including cabbage and spinach to estimate the concentration of glutathione using HPLC. The research was carried out to determine how much glutathione was present in the vegetables. The glutathione concentration in cabbage leaves was 0.84 µmol/g, which was much higher than the glutathione concentration in spinach leaves, which was 1.5 µmol/g. The percentage of oxidized glutathione and cysteine found in dark green leafy vegetables was significantly higher than the percentage of reduced glutathione, and these vegetables also contained a significant amount of the reduced amino acid cysteine. In addition, the findings of the current research were in agreement with those of Nacca et al. (2021), who determined the length of time required for glutathione to be retained in one of the various seeds of the ecotype of *Brassica rapa*. The length of time required for glutathione to be retained was 4.8 min when measured using an HPLC instrument. This difference in the glutathione concentrations was caused by the different times in which the raw material was obtained during the different stages of the work, as well as the fact that there was no knowledge of the differences in the time or conditions of storage and the changes that it went through at that time and transportation conditions, as well as the possibility of exposure to high or low temperatures, drought, and chemicals that were used. In addition, there was no knowledge of the differences in the time or conditions of storage and the changes that it went through at that (Alscher, 1989, Mills et al., 1997).

### 4.4 Physical and chemical properties of glutathione-incorporated meat patties

#### 4.4.1 pH

The disintegration of proteins and the separation of the amine group may be two of the variables that are responsible for the rise in pH levels during the storage period. In the present investigation, these changes may have occurred as a result of the influencing factors that occurred throughout the storage period (Hashim and Fadhil, 2021). The rise in the number of microorganisms that develop throughout storage is another factor that contributes to the problem. These microorganisms break down proteins, which causes the formation of volatile bases and an increase in pH (Aslam et al., 2020). The addition of peptides to meat can improve its quality in several ways, most notably the pH levels and other physical characteristics.

#### 4.4.2 Water holding capacity

When it comes to meat products, one of the quality marks that must be met is known as the water-holding capacity (WHC). The greater the value of this quality mark, the more the consumer will like and appreciate the product. An increase in the WHC of the meat patties was accomplished by adding more glutathione to the mixture. Because it impacts other properties such as appearance, organoleptic characteristics, and meat quality, as the known fact that WHC is one of the most important quality criteria of fresh meat (Das et al., 2011). Because of its low molecular weight, meat that has been treated with biological peptides has a high WHC. This is because it is more successful than other substances in retaining water, and the concentration of the peptides affects the WHC. The extensive hydrolysis that occurs throughout the storage period is the cause of the reduction in the WHC of the meat, and the subsequent degradation of the proteins brings about a reduction in the WHC of the proteins (Taheri et al., 2013, Halim et al., 2016). There is a possibility that this is related to the composition of the muscle protein as well as the pH of the meat (Ibrahim et al., 2018). Furthermore, in general, the WHC of the meat decreases with the increasing length of time spent in storage.

#### 4.4.3 Extract release volume

It is regarded as one of the meat’s physical characteristics, and its relationship to the meat’s capacity to hold water is shown by the volume of the liquid that is extracted from the flesh. The length of time the meat patties were stored also affected the results, as the amount of liquid that was extracted from them became more volume as the length of time they were stored rose, up to the point where they reached the end of the storage stage. Meat patties required a significant amount of moisture to be drained before they could be prepared. The poor WHC as a result of the loss that happened during storage is the reason there was a rise in the volume of the extracted liquid during the storage period. This loss was the cause of the increase in volume (Al-Baydani, 2020).

#### 4.4.4 Free fatty acids

The measurement of free fatty acids often referred to as FFAs, is commonly used as a measure for fat oxidation since they are one of the compounds that result from the breakdown of fat (Verma and Srivastav, 2017). This was in comparison to the two meat patties that were given the control treatment, which both showed a higher percentage of FFAs. Additionally, the results showed that there were differences between the values of FFAs in different concentrations and storage durations. According to the findings presented in Fig. 7(D), we observe that the values of FFAs for meat patties that were treated with glutathione derived from the sources utilized and at each of the concentrations utilized converged to the same point. The activity of the enzyme lipase, which converts triglycerides into FFAs while the product is stored, is responsible for the increase in the percentage of FFAs found in meat patties that have been frozen or refrigerated for a longer period. FFAs are one of the products of the hydrolysis of fats caused by this enzyme and other types of lipolytic bacteria (Youssef, 2014). The enzyme hydrolase, which is responsible for the release of FFAs during storage, is one of the targets of glutathione, which inhibits its activity. As a result, the proportion of FFAs decreases when the concentration of glutathione does as well (Wu, 2018).

#### 4.4.5 Peroxide value

The peroxide values, also known as PVs, are an expression of the amount of oxidation that has occurred in the oil during its early stages of oxidation and represents the peroxide content of the oil (Nayak et al., 2016). The results demonstrate that there are differences between the peroxide values and of the meat patties added to glutathione in different concentrations and times. These values were lower when compared to the control treatment, which had values that reached 4.74 mEq/kg at the end of the storage phase, as shown in the same Fig. 7(E). The concentration of glutathione affected the peroxide values of meat patties, as we found that an increase in the added concentration resulted in a drop in the peroxide values. The antioxidant activity of the peptides may be responsible for the reduction in peroxide levels that were observed in the meat patties that had been treated with them. This was noticed through research into the antioxidant activity of the peptides (Al-Halfi, 2016). The low levels of peroxide value may be caused by the capacity of glutathione to alter the structure of fat by lowering the rate of activity of fat enzymes known as fat synthase (Wu, 2018). The production of peroxides and heme proteins like myoglobin and hemoglobin are among the primary catalysts for the initiation of the oxidation process. The oxidation of fats in meat is caused by iron's role as a primary catalyst for oxidation (Min and Ahn, 2005).

#### 4.4.6 Thiobarbituric acid

Malondialdehyde is regarded as one of the most significant aldehydes formed during the oxidation of lipids in unsaturated fatty acids. It is of utmost significance in meat because even trace levels of it can induce rancidity and aromas that are unappealing (Jones, 2017, Verma and Srivastav, 2020a). When compared with the control treatment, the acid values of the beef patties that had been treated with glutathione showed a significant drop. This was because the findings revealed that there were changes in the concentrations and durations of TBA values. The control treatment was the one that produced glutathione from spinach leaves and red cabbage leaves. As can be seen in Fig. 7(F), the acid values go down when there is a higher quantity of glutathione added to the mixture. The continuous production of aldehydes during the storage period is responsible for the elevated levels of TBA that were found across all of the treatments, notably in the control group (Chauhan et al., 2018). The elevated acid levels may be the result of oxidation that had been placed throughout storage. Oxidation of triglycerides at the double bonding sites is something that might take place after some time has passed during storage (Sohaib et al., 2017). While the drop in TBA values can be attributed to the capacity of physiologically active peptides to lower oxidative stress via hydrogen donation from amino acids to interrupt the oxidation chain reaction, this ability is not entirely responsible for the drop (Aslam et al., 2020).

## 5 Conclusion

The findings of the study lead us to the conclusion that there is no cause for concern regarding the level of glutathione found in food. It was satisfactory in spinach leaves and red cabbage leaves compared to other plants, and adding glutathione in different concentrations led to an increase in the shelf life of each of the cold-stored minced meat patties. This was because glutathione inhibits fat oxidation and increases the shelf life of foods. Additionally, it was found to be a risk-free replacement for conventional antioxidants used in industry. It did not have any negative side effects, and by boosting the concentration of glutathione, it enhanced the body's natural defenses against the creation of free radicals, which can in turn stop fat from being oxidized. As a consequence of this, these glutathione compounds have shown that they have a powerful ability, which may be attributed to the numerous features that they possess. This glutathione had the potential to serve as a viable candidate for the development of functional foods. This was because glutathione has been shown to have a variety of health benefits, including antioxidant activity. Therefore, glutathione from spinach and red cabbage leaf extract might well be employed and valorized by including it in a variety of food formulations to boost product quality and ensure the longevity of the food processing sector. The hypothesis also held that this glutathione had beneficial properties and no toxicity. According to the results of the current research, glutathione derived from leaf extract has the potential to be used in the production of other food products other than functional foods. Researchers and scientists that are affiliated with the food industry and are seeking innovative food products that may assure food safety and security as well as manage and improve overall human health may find this to be a valuable therapeutic and nutritional strategy. As a consequence of this, we can draw the following conclusion based on the findings of the current investigation, which are discussed in the lines that came before this one: There are enormous opportunities for additional research, which include determining the properties of preservation, the safety aspects, and the physiological benefits of glutathione that are derived from the leaf extracts of spinach and red cabbage. In addition, the current study recommends to researchers and scientists that investigations into the nutritional, organoleptic, and other vital qualities of the developed food formulations be conducted to guarantee that consumers would purchase and use the products.

## Declaration of Competing Interest

The authors declare that they have no known competing financial interests or personal relationships that could have appeared to influence the work reported in this paper.
